# Supplementary material for: The Duration of Increased Grain Feeding Affects the Microbiota throughout the Digestive Tract of Yearling Holstein Steers
Source: Microorganisms. 2020 Nov 25;8(12):1854. doi: 10.3390/microorganisms8121854 (PMC7761415; doi:10.3390/microorganisms8121854)
Supplement: Supplementary file 1 [file microorganisms-08-01854-s001.pdf]

microorganisms

MDPI

Supplementary

Table S1. Chemical composition of dietary ingredients.

	**Grass hay**	**Barley Grain**	Vitamin/Mineral pellets
DM, %	95.5	92.9	95.3
CP, % DM	9.9	7.2	36.7
NDF, % DM	61.0	18.5	10.0
ADF, DM	38.2	6.7	4.5
Starch, DM	2.4	59.0	26.8
Ca, DM	0.67	0.07	2.51
P, DM	0.13	0.41	0.42
Mg, DM	0.17	0.15	1.65
K, DM	1.50	0.63	3.31
Na, DM	1.50	0.01	1.12

Table S2. Ingredients composition of nutrient pellets (%).

Ground barley	40.5
Soybean meal	35
Urea	5.4
Limestone	5.1
Salt	2.2
Tallow	1
Dynamate [1]	10.2
Magnesium Oxide	0.29
Selenium 2000	0.18
EDDI[2]	0.07
Potassium Chloride	0.06
Vitamin A 1000	0.003
Manganese Oxide	0.04
Vitamin D 500	0.003
Copper sulfate	0.001
EDDI [2]	0.001
Zinc Oxide	0.001
Ferrous Carbonate	0.001

[1] Feed-grade potassium sulfate and magnesium sulfate, [2] Ethylenediamine dihydroiodide.

Table S3. Mapping file for study on the duration of moderate grain feeding (Treat.: 0 = no grain feeding, 7 moderate grain feeding for 7 d, 21 = moderate grain feeding for 21 days) on microbiota at different regions of the digestive tract (Rumen, Jejunum, Ileum, Cecum, Colon, and Rectum).

Sample ID	Barcode Sequence	Linker Primer Sequence	Region	Treat.
1.ca	GCCTGTCTGCAA	TATGGTAATTGTGTGCCAGCMGCCGCGGTAA	Cecum	0
2.ca	GTCGAATTTGCG	TATGGTAATTGTGTGCCAGCMGCCGCGGTAA	Cecum	0
3.ca	GCATCAGAGTTA	TATGGTAATTGTGTGCCAGCMGCCGCGGTAA	Cecum	0
4.ca	GTGGTCATCGTA	TATGGTAATTGTGTGCCAGCMGCCGCGGTAA	Cecum	0
5.ca	CTGAAGGGCGAA	TATGGTAATTGTGTGCCAGCMGCCGCGGTAA	Cecum	0
6.ca	CTATCATCCTCA	TATGGTAATTGTGTGCCAGCMGCCGCGGTAA	Cecum	7
7.ca	AGCTGCACCTAA	TATGGTAATTGTGTGCCAGCMGCCGCGGTAA	Cecum	7
8.ca	ACTCTAGCCGGT	TATGGTAATTGTGTGCCAGCMGCCGCGGTAA	Cecum	7
9.ca	ACCGTGCTCACA	TATGGTAATTGTGTGCCAGCMGCCGCGGTAA	Cecum	7
10.ca	AGGTCCAAATCA	TATGGTAATTGTGTGCCAGCMGCCGCGGTAA	Cecum	7
11.ca	TATGCCAGAGAT	TATGGTAATTGTGTGCCAGCMGCCGCGGTAA	Cecum	21
12.ca	TATCACCGGCAC	TATGGTAATTGTGTGCCAGCMGCCGCGGTAA	Cecum	21
13.ca	GAGACGTGTTCT	TATGGTAATTGTGTGCCAGCMGCCGCGGTAA	Cecum	21
14.ca	AGATGATCAGTC	TATGGTAATTGTGTGCCAGCMGCCGCGGTAA	Cecum	21
15.ca	GCAGATTTCCAG	TATGGTAATTGTGTGCCAGCMGCCGCGGTAA	Cecum	21
1.col	ACTGACTTAAGG	TATGGTAATTGTGTGCCAGCMGCCGCGGTAA	Colon	0
2.col	GATGCTGCCGTT	TATGGTAATTGTGTGCCAGCMGCCGCGGTAA	Colon	0
3.col	ACAGCTCAAACA	TATGGTAATTGTGTGCCAGCMGCCGCGGTAA	Colon	0
4.col	GAGCGTATCCAT	TATGGTAATTGTGTGCCAGCMGCCGCGGTAA	Colon	0
5.col	TGGCTTTCTATC	TATGGTAATTGTGTGCCAGCMGCCGCGGTAA	Colon	0
6.col	CCTTGACCGATG	TATGGTAATTGTGTGCCAGCMGCCGCGGTAA	Colon	7
7.col	CGATAGGCCTTA	TATGGTAATTGTGTGCCAGCMGCCGCGGTAA	Colon	7
8.col	CTCCCTTTGTGT	TATGGTAATTGTGTGCCAGCMGCCGCGGTAA	Colon	7
9.col	CTTAGGCATGTG	TATGGTAATTGTGTGCCAGCMGCCGCGGTAA	Colon	7
10.col	AATGACCTCGTG	TATGGTAATTGTGTGCCAGCMGCCGCGGTAA	Colon	7
11.col	CGTAGGTAGAGG	TATGGTAATTGTGTGCCAGCMGCCGCGGTAA	Colon	21
12.col	AACCAAACTCGA	TATGGTAATTGTGTGCCAGCMGCCGCGGTAA	Colon	21
13.col	ACCTTACACCTT	TATGGTAATTGTGTGCCAGCMGCCGCGGTAA	Colon	21
14.col	CATCATACGGGT	TATGGTAATTGTGTGCCAGCMGCCGCGGTAA	Colon	21
15.col	TACGGATTATGG	TATGGTAATTGTGTGCCAGCMGCCGCGGTAA	Colon	21
1.fe	ATTAAGCCTGGA	TATGGTAATTGTGTGCCAGCMGCCGCGGTAA	Rectum	0
2.fe	TTCCTAGGCCAG	TATGGTAATTGTGTGCCAGCMGCCGCGGTAA	Rectum	0
3.fe	CCAGATATAGCA	TATGGTAATTGTGTGCCAGCMGCCGCGGTAA	Rectum	0
4.fe	GGTCTCCTACAG	TATGGTAATTGTGTGCCAGCMGCCGCGGTAA	Rectum	0
5.fe	GCCGTCTCGTAA	TATGGTAATTGTGTGCCAGCMGCCGCGGTAA	Rectum	0
6.fe	ATGGGCGAATGG	TATGGTAATTGTGTGCCAGCMGCCGCGGTAA	Rectum	7
7.fe	TAACGCTGTGTG	TATGGTAATTGTGTGCCAGCMGCCGCGGTAA	Rectum	7
8.fe	ATAGCGAACTCA	TATGGTAATTGTGTGCCAGCMGCCGCGGTAA	Rectum	7
9.fe	GATCTCTGGGTA	TATGGTAATTGTGTGCCAGCMGCCGCGGTAA	Rectum	7
10.fe	AACCGCATAAGT	TATGGTAATTGTGTGCCAGCMGCCGCGGTAA	Rectum	7
11.fe	GCGTTGCAAACT	TATGGTAATTGTGTGCCAGCMGCCGCGGTAA	Rectum	21
12.fe	ACGTGTAGGCTT	TATGGTAATTGTGTGCCAGCMGCCGCGGTAA	Rectum	21
13.fe	GAGAGTCCACTT	TATGGTAATTGTGTGCCAGCMGCCGCGGTAA	Rectum	21
14.fe	CTGGGTATCTCG	TATGGTAATTGTGTGCCAGCMGCCGCGGTAA	Rectum	21
15.fe	GAACGGGACGTA	TATGGTAATTGTGTGCCAGCMGCCGCGGTAA	Rectum	21
1.IL	GACTACCCGTTG	TATGGTAATTGTGTGCCAGCMGCCGCGGTAA	Ileum	0
2.IL	AAGACAGCTATC	TATGGTAATTGTGTGCCAGCMGCCGCGGTAA	Ileum	0
3.IL	GTTCGGTGTCCA	TATGGTAATTGTGTGCCAGCMGCCGCGGTAA	Ileum	0
4.IL	TTCGATGCCGCA	TATGGTAATTGTGTGCCAGCMGCCGCGGTAA	Ileum	0
5.IL	ACTGATGGCCTC	TATGGTAATTGTGTGCCAGCMGCCGCGGTAA	Ileum	0
6.IL	CGCTCACAGAAT	TATGGTAATTGTGTGCCAGCMGCCGCGGTAA	Ileum	7
7.IL	GACTTCATGCGA	TATGGTAATTGTGTGCCAGCMGCCGCGGTAA	Ileum	7
8.IL	ATTCGGTAGTGC	TATGGTAATTGTGTGCCAGCMGCCGCGGTAA	Ileum	7

9.IL	CGAGCTGTTACC	TATGGTAATTGTGTGCCAGCMGCCGCGGTAA	Ileum	7
10.IL	CAACACATGCTG	TATGGTAATTGTGTGCCAGCMGCCGCGGTAA	Ileum	7
11.IL	ATTCTCTCACGT	TATGGTAATTGTGTGCCAGCMGCCGCGGTAA	Ileum	21
12.IL	CGACTCTAAACG	TATGGTAATTGTGTGCCAGCMGCCGCGGTAA	Ileum	21
13.IL	GTCTTCAGCAAG	TATGGTAATTGTGTGCCAGCMGCCGCGGTAA	Ileum	21
15.IL	AGGGTGACTTTA	TATGGTAATTGTGTGCCAGCMGCCGCGGTAA	Ileum	21
1.Jej	ATCCCTACGGAA	TATGGTAATTGTGTGCCAGCMGCCGCGGTAA	Jejunum	0
2.Jej	CACCCGATGGTT	TATGGTAATTGTGTGCCAGCMGCCGCGGTAA	Jejunum	0
3.Jej	TACACAAGTCGC	TATGGTAATTGTGTGCCAGCMGCCGCGGTAA	Jejunum	0
4.Jej	GTGTTCCCAGAA	TATGGTAATTGTGTGCCAGCMGCCGCGGTAA	Jejunum	0
5.Jej	TTCTGAGAGGTA	TATGGTAATTGTGTGCCAGCMGCCGCGGTAA	Jejunum	0
6.Jej	TTCTCCATCACA	TATGGTAATTGTGTGCCAGCMGCCGCGGTAA	Jejunum	7
7.Jej	ATTTAGGACGAC	TATGGTAATTGTGTGCCAGCMGCCGCGGTAA	Jejunum	7
8.Jej	CCGAGGTATAAT	TATGGTAATTGTGTGCCAGCMGCCGCGGTAA	Jejunum	7
9.Jej	AACTTTCAGGAG	TATGGTAATTGTGTGCCAGCMGCCGCGGTAA	Jejunum	7
10.jej	TGGTTGGTTACG	TATGGTAATTGTGTGCCAGCMGCCGCGGTAA	Jejunum	7
11.Jej	GGATAGCCAAGG	TATGGTAATTGTGTGCCAGCMGCCGCGGTAA	Jejunum	21
12.Jej	TGCACGTGATAA	TATGGTAATTGTGTGCCAGCMGCCGCGGTAA	Jejunum	21
13.Jej	ATTGACCGGTCA	TATGGTAATTGTGTGCCAGCMGCCGCGGTAA	Jejunum	21
14.Jej	TGTGGCTCGTGT	TATGGTAATTGTGTGCCAGCMGCCGCGGTAA	Jejunum	21
15.Jej	GTCGTCCAAATG	TATGGTAATTGTGTGCCAGCMGCCGCGGTAA	Jejunum	21
1.RF	GCCGTAAACTTG	TATGGTAATTGTGTGCCAGCMGCCGCGGTAA	Rumen	0
2.RF	ATTGTTCCTACC	TATGGTAATTGTGTGCCAGCMGCCGCGGTAA	Rumen	0
3.RF	GCCACGACTTAC	TATGGTAATTGTGTGCCAGCMGCCGCGGTAA	Rumen	0
4.RF	AGACAGTAGGAG	TATGGTAATTGTGTGCCAGCMGCCGCGGTAA	Rumen	0
5.RF	GGTTTAACACGC	TATGGTAATTGTGTGCCAGCMGCCGCGGTAA	Rumen	0
6.RF	CCTGTCCTATCT	TATGGTAATTGTGTGCCAGCMGCCGCGGTAA	Rumen	7
7.RF	AGGTGAGTTCTA	TATGGTAATTGTGTGCCAGCMGCCGCGGTAA	Rumen	7
8.RF	CTCGTGAATGAC	TATGGTAATTGTGTGCCAGCMGCCGCGGTAA	Rumen	7
9.RF	AGCGTAATTAGC	TATGGTAATTGTGTGCCAGCMGCCGCGGTAA	Rumen	7
10.RF	GCGTCCATGAAT	TATGGTAATTGTGTGCCAGCMGCCGCGGTAA	Rumen	7
11.RF	CAACGTGCTCCA	TATGGTAATTGTGTGCCAGCMGCCGCGGTAA	Rumen	21
12.RF	GGAATCCGATTA	TATGGTAATTGTGTGCCAGCMGCCGCGGTAA	Rumen	21
13.RF	GTCGCCGTACAT	TATGGTAATTGTGTGCCAGCMGCCGCGGTAA	Rumen	21
14.RF	GTAATGCGTAAC	TATGGTAATTGTGTGCCAGCMGCCGCGGTAA	Rumen	21
15.RF	GGTTCCATTAGG	TATGGTAATTGTGTGCCAGCMGCCGCGGTAA	Rumen	21

Table S4. Relative abundances of abundant phyla (>1%) for each treatment (D0 = no grain feeding, D7 moderate grain feeding for 7 d, D21 = moderate grain feeding for 21 d.

Phylum		Rumen			
	D0	D7	D21	SE	Significance, *p-values*
Bacteroidetes	35.8b	42.4^{a}	35.7^{b}	1.64	<0.01
Firmicutes	60.3^{a}	50.9^{b}	56.4^{c}	1.79	<0.01
Proteobacteria	<1	2.4	1.7	0.77	>0.10
Tenericutes	<1	<1	1		
Actinobacteria	2.4	3.3	2.2	0.49	>0.10
Fibrobacteres	<1	2.0	1.6	0.6	
		Jejunum			
	D0	D7	D21		
Bacteroidetes	3.6	1	2.8		
Firmicutes	68.4^{ab}	74.5^{a}	61.7^{b}	6.08	<0.05
Proteobacteria	3.7^{b}	6.4^{ab}	8.9^{a}	1.77	<0.05
Actinobacteria	9.9	9.1^{b}	14.5	2.96	>0.10
Verrucomicrobia	<1	<1	1		
Euriarchiota	14.3	8.5	11.6	3.43	>0.10
		Ileum			
	D0	D7	D21		
Firmicutes	88.3^{ab}	92.7^{a}	81.9^{b}	4.74	<0.05
Proteobacteria	1.0	<1	10.6		
Actinobacteria	5.5^{b}	3.7^{b}	11.9^{a}	1.26	<0.01
Euriarchiota	5.7^{a}	2.9^{b}	4.7^{ab}	1.37	<0.05
Phylum		Cecum			
	D0	D7	D21		
Bacteroidetes	40.7	442	41.2	4.75	>0.10
Firmicutes	55.1	51.7	52.0	5.06	>0.10
Spirochaetes	2.3	1.7	1.9	0.22	>0.10
Tenericutes	1		1	0.24	>0.10
Verrucomicrobia	1	1	1	0.05	>0.10
		Colon			
	D0	D7	D21		
Bacteroidetes	42.5	39.6	41.8	3.92	>0.10
Firmicutes	55.1^{ab}	58.2^{a}	50.6^{b}	2.63	<0.05
Spirochaetes	1b	1.8ab	4.3a	0.45	<0.05
Tenericutes	1	<1	1		
Actinobacteria	<1	<1	1		
		Feces			
	D0	D7	D21		
Bacteroidetes	41.7^{a}	40.0^{ab}	37.2^{b}	2.13	<0.05
Firmicutes	57.1	56.5	59.0	1.82	>0.10
Spirochaetes	<1	2.1	1.9	0.13	>0.10
Tenericutes	1	<1	<1	0.10	
Verrucomicrobia	<1	1	1	0.03	>0.10

[a,b,c] Means in a row with a different superscripts differ($p < 0.05$).
